# Deciphering the tumor ecosystem dynamics undergoing immunochemotherapy therapy across multiple cancer types unveils the immunosuppressive role of S100A4 in fibroblasts by promoting PD-L1 expression in tumor cells

**DOI:** 10.3389/fcell.2025.1613296

**Published:** 2025-07-23

**Authors:** Bo Yang, Ruiji Chen, Mali Zu, Jie Yao, Hong Ren, Yingxue Lin, Bo Zhang, Tianjiao Ji, Yang Liu

**Affiliations:** ^1^ Department of Thoracic Surgery, The First Medical Center of the People Liberty Army General Hospital, Beijing, China; ^2^ Postgraduate School, Medical School of Chinese People Liberty Army, Beijing, China; ^3^ Department of Thoracic Surgery, Hainan Hospiotal of the People Liberty Army General Hospital, Sanya, Hainan, China; ^4^ CAS Key Laboratory for Biomedical Effects of Nanomaterials and Nanosafety, CAS Center for Excellence in Nanoscience, National Center for Nanoscience and Technology, Beijing, China; ^5^ Department of General Surgery, Jinling Hospital, Affiliated Hospital of Medical School, Nanjing University/General Hospital of Eastern Theater Command, PLA, Nanjing, Jiangsu, China; ^6^ The School of Medicine, Nankai University, Tianjin, China; ^7^ Department of Cardiothoracic Surgery, The 80Th Group Army Hospital of Chinese People Liberty Army, Weifang, Shandong, China

**Keywords:** neoadjuvant therapy, cross-cancer atlas, tumor microenvironment, single-cell RNA sequencing, S100A4

## Abstract

**Background:**

Neoadjuvant therapy (NAT) has transformed cancer treatment by improving surgical outcomes and survival rates, yet resistance mechanisms across multiple cancer types remain unclear. This study aimed to decipher tumor ecosystem dynamics during NAT using cross-cancer single-cell sequencing data, focusing on identifying key mediators of immunosuppression and treatment resistance.

**Methods:**

Single-cell RNA-sequencing (scRNA-seq) datasets from five solid tumors (esophageal squamous cell carcinoma, esophagogastric junction carcinoma, colorectal cancer, cervical cancer, and triple-negative breast cancer) were integrated. The data from these five cancer types underwent a rigorous process to standardize cell types across all datasets. Cell-cell communication analysis, Meta-Programs (MPs) via non-negative matrix factorization, and functional enrichment were performed. Immunohistochemistry (IHC) and Western blot validated S100A4 expression and PD-L1 induction *in vitro*.

**Results:**

We constructed a single-cell map across cancer types and systematically characterized dynamic changes in tumor cells and diverse microenvironmental cell populations following neoadjuvant therapy, along with thier gene expression and pathway alterations. Our findings highlight that crosstalk between cancer-associated fibroblasts (CAFs) and tumor cells represents a critical determinant of neoadjuvant therapy resistance. Fibroblasts underwent significant state transitions post-treatment, marked by hypoxia-associated gene upregulation (e.g., *S100A4*) and immunosuppressive pathways. Meta-Programs (MPs) analysis identified a hypoxia-driven fibroblast state (MP5) containing *S100A4* that correlated with treatment resistance. *In vitro* experiments, *S100A4* co-localized with α-SMA + fibroblasts and directly induced PD-L1 expression in tumor cells, linking CAFs secreted *S100A4* to immunosuppressive PD-L1 upregulation.

**Conclusion:**

This cross-cancer single-cell atlas reveals S100A4, secreted by CAFs, as a conserved mediator of PD-L1 upregulation in tumor cells, driving immunosuppression and resistance to nICT. The atlas and mechanistic findings provide a rationale for targeting S100A4 to enhance treatment efficacy, pending validation in larger cohorts and mechanistic studies. This resource also supports the development of personalized, cross-cancer neoadjuvant strategies.

## Background

Neoadjuvant therapy (NAT) exhibits significant advantages in cancer treatment, notably in reducing tumor size, enhancing surgical feasibility, and potentially eliminating small metastases (Jiang et al., 2023). Particularly, neoadjuvant immunochemotherapy (nICT) has yielded impressive clinical outcomes, revolutionizing the treatment landscape for many solid tumors (Zheng et al., 2023; Huang et al., 2023; Kanani et al., 2021; Sun et al., 2024). For example, on esophageal cancer, nICT significantly increases the down-stage rate, making more patients eligible for radical resection, and improves the 3-year overall survival rate by 15% (Duan et al., 2022). Nevertheless, due to the inter- and intra-tumor heterogeneity, not all patients respond optimally to NAT. Thus, gaining a deeper understanding of how NAT impacts the cellular composition of tumors, and their microenvironment is crucial for understanding drug resistance and optimizing treatment strategies.

The tumor microenvironment (TME) is a complex ecosystem comprising tumor cells, immune cells, extracellular matrix, secretions, and lymphatic vascular networks (Xiao and Yu, 2021). The cellular and molecular components of the TME are closely linked to treatment efficacy (Bagaev et al., 2021). The remodeling of tumor ecosystem was considered to play critical roles in progression and drug resistance of multiple tumors (Yang et al., 2020; Manabe and Bivona, 2022; Chen et al., 2023; Luo et al., 2023). Such dynamic process relies on complex intercellular interactions between tumor cells and diverse TME cells, such as cancer-associated fibroblasts and immune cells (Mao et al., 2021). Recent technological breakthroughs in RNA sequencing (scRNA-seq) have facilitated high-resolution analysis of tumor ecosystem atlases of cancer, serving as a potent tool for elucidating the biological properties and dynamics of cells during the neoadjuvant therapy. Prior single-cell transcriptome analyses have highlighted the presence of marked immunosuppressive features in the TME of tumors with suboptimal therapeutic outcomes (Han et al., 2023; Li et al., 2024; Sathe et al., 2023). However, most of these single-cell studies have focused on specific cancer types, preventing comprehensive and in-depth analysis of the tumor ecosystem dynamics across cancer. In addition, there are personalized cell subset annotation for each study, which makes cross-research between cancer types difficult, and whether a cell subset defined in one cancer type can be identified in other cancer types remains to be determined. Therefore, it is needed to first establish an integrated cross-cancer single cell map to facilitate the exploration of consensus regulatory programs during neoadjuvant therapy.

In this study, we integrated multiple single-cell datasets covering five solid tumor types from patients undergoing NAT, for which pre- and post-treatment samples were involved. Based on the integrated single cell atlas, we systematically characterized the effects of neoadjuvant therapy on cellular composition and the biological changes of different cell types before and after treatment. Through cell interaction analysis, we identified the potential molecular interactions between tumor cells and TME cells that related to treatment response and performed further experimental validation. Our findings not only reveal the consensus cellular and molecular dynamics during neoadjuvant therapy but also provide insights for the development of cross-cancer immunotherapy.

## Methods

### Data accessing for scRNA-seq data

The single-cell RNA sequencing (scRNA-seq) data utilized in this study were exclusively sourced from the Gene Expression Omnibus (GEO) database in the United States. These datasets can be accessed and downloaded using their respective GEO Series accession numbers (GSE numbers). This study encompasses cervical cancer (CC, GSE236738), colorectal cancer (CRC, GSE205506), esophagogastric junction cancer (EGJC, GSE244739), esophageal squamous cell carcinoma (ESCC, GSE221561), and triple-negative breast cancer (TNBC, GSE263995). Cell annotation metadata were obtained from the corresponding publications or Supplementary Material.

### scRNA-seq data pre-processing and quality control

Following the download of raw data from the GEO database, we processed the scRNA-seq data utilizing the Seurat R package (version 5.1.0) to convert it into Seurat objects with filtering parameters set at min.cells = 3 and min.features = 200 within the CreateSeuratObject () function (Hao et al., 2024). Subsequently, quality control was conducted on each Seurat object derived from different cancer types. High-quality cells were retained based on specific criteria: cells expressing more than 200 genes; mitochondrial transcript percentages below 10%; and UMI counts ranging between 2,000 and 25,000.

### Normalization, dimensionality reduction and clustering

The scRNA-seq count matrix was normalized using the SCTransform () function in Seurat with default parameters. The top 3,000 variable genes were selected for principal component analysis (PCA). To reduce dimensionality among cells, PCA was performed utilizing these top variable genes. Batch effects were mitigated through application of the R package Harmony (version 1.2.1) (Korsunsky et al., 2019), which iteratively corrected PCA embeddings based on the top 30 PCA components while setting group.by.vars to samples. The resulting harmonized PCA components were subsequently employed for cell clustering as well as non-linear dimensionality reduction via Uniform Manifold Approximation and Projection (UMAP) (McInnes et al., 2020).

For the cell clustering of each cancer scRNA dataset, we initially constructed a KNN graph based on the Euclidean distance in harmonized PCA space. This step was executed using the FindNeighbors () function, which takes as input the previously defined dimensionality of the dataset (Top 30 Dims). Subsequently, we applied modularity optimization techniques, specifically employing the Louvain algorithm as default method, to iteratively group cells with the objective of optimizing the standard modularity function. The FindClusters () function was employed to carry out this procedure, with the resolution parameter set at 0.5, leading to the formation of seven distinct cell clusters. These clusters align precisely with the cell types identified by the original authors (Yang et al., 2023), namely, epithelial cells, fibroblasts, macrophages, mast cells, T cells, B cells, and endothelial cells.

### Integrating single-cell atlases and cell annotations

First, perform integration operations on five single-cell datasets. Merge all datasets into a single object using the merge () function, group them by cancer type, and sequentially conduct SCTransform normalization, principal component analysis (PCA) for dimensionality reduction, and Harmony batch effect correction. Finally, complete the joint clustering analysis using Seurat v4.0 software to generate an initial integrated atlas. Identify the marker genes of each cell cluster using the FindAllMarkers () function and compare them with known cell markers in the database (Hu et al., 2023; Franzén et al., 2019) to complete the cell annotation.

In the initial integrated atlas, there are discrepancies between the single-cancer annotation and the integrated annotation for some cells. For example, some cells in colorectal cancer (CRC) that were individually annotated as B cells were re-annotated as mast cells in the integrated atlas. To improve the accuracy of cell annotation, use the initial integrated atlas as a reference benchmark and perform mapping operations on each of the five single-cell datasets. Use the FindTransferAnchors () function with parameters set as dims = 30 and reference.reduction = “PCA” to identify the anchors between the initial integrated dataset and the Seurat objects of other cancer types. After determining the anchors, use the TransferData () function to compare the cell types annotated in single-cancer datasets based on the cell annotation results of the integrated atlas. The TransferData () function returns a matrix containing predicted cell types and prediction scores (Hao et al., 2021), which is then integrated into the metadata. By analyzing the prediction score matrix and cell annotation discrepancies, cells with prediction scores below 0.8 are screened out and identified as abnormal cells with annotation differences. Specifically, 4821 cells (15.2%) were filtered out from the CC dataset, 2034 cells (8%) from the CRC dataset, 3596 cells (13.1%) from the EFJC dataset, 4469 cells (9%) from the ESCC dataset, and 733 cells (6.6%) from the TNBC dataset.​

Finally, after removing the abnormal cell data, re-perform the SCTransform normalization, PCA dimensionality reduction, Harmony batch effect correction, clustering analysis, and cell annotation processes on the filtered datasets to construct the final integrated single-cell atlas. Use Seurat tools in combination with the R package ggplot2 (version 3.5.1) for the visualization analysis of scRNA-seq data (Ginestet, 2011). Generate UMAP plots to display the differences in cell abundance among different cancer groups; meanwhile, to ensure data comparability, perform downsampling on the number of cells within each group to make the cell numbers of all groups reach the same level.

### Identification and functional analysis of differentially expressed genes

The FindAllMarkers () function in Seurat was used to identify cell-type-specific genes and differentially expressed genes between treated and baseline samples with default parameters. P-values were adjusted using the Bonferroni test. Most marker genes and differentially expressed genes are provided in Supplementary Tables S2, S4. For genes potentially related to the progression of neoadjuvant therapy, we described the fold changes and adjusted P-values in the Results section. The R package clusterProfiler (version 4.12.6) was applied to perform pathway enrichment analysis of the identified gene sets (Wu et al., 2021).

### Enrichment of signaling pathway

For the integrated scRNA-seq data, genes were pre-ranked by standardized variance and then enriched using Gene Set Enrichment Analysis (GSEA) (Subramanian et al., 2005). The Gene Ontology (GO) knowledgebase and The Kyoto Encyclopedia of Genes and Genomes (KEGG) were served as the gene sets database (Ontology Consortium et al., 2023; Kanehisa et al., 2017). Pathways/terms with an adjusted p-value greater than 0.05 were filtered out, and we focused on pathways with an overlap of more than 10 genes. Pathways associated with the efficacy of neoadjuvant therapy were described in the Results section.

### Identifying cell consistency status using cNMF

Non-negative matrix factorization (NMF) is well-suited for decomposing scRNA-seq data, effectively reducing large complex matrices into interpretable gene programs. We used the GeneNMF R package (version 0.4.0) for NMF analysis of the integrated single-cell data (Yerly et al., 2024). The RunNMF() function can be directly applied to a Seurat object, and it saves the NMF results as a new dimensionality reduction. To identify robust programs, we applied the multiNMF() function across multiple values of (k) (from 6 to 9) and determined programs that were consistently found across these runs. We then combined the gene programs identified across multiple samples into metaprograms (MPs). Based on the Jaccard index, we cut the similarity tree at a specified height to identify blocks of similar programs and derive consensus gene signatures for each MP.

To better understand the functions of these MPs, we compared them to known signatures from public databases. The runGSEA () function was used to scan the MSigDB and evaluate the overlap between the detected MPs and the signatures in the databases (Canzler and Hackermüller, 2020; Liberzon et al., 2015). In addition, summarized MPs were downloaded from the 3CA database (Tyler et al., 2024) for reference to further confirm the universality and robustness of the MPs identified in this study. By calculating the Jaccard index between public MPs and the identified MPs, the maximum value was used as the basis for annotating the identified MPs. The analysis of the protein interaction network between genes in the MPs was completed through the STRING database (Szklarczyk et al., 2023).

### Cell-cell interaction and ligand-receptor analysis

We investigated the cell-cell interactions among diverse cell types using the R package CellChat (v1.1.3) (Jin et al., 2025), which could quantitatively infer and analyze intercellular communication networks from scRNA-seq data. We mainly utilized the “netVisual_circle” function of CellChat, and the corresponding results were presented via crosstalk maps and ligand-receptor heatmaps.

### Bulk RNA-seq analysis

The counts and transcripts per million matrix of gene expression were obtained from the study by Zhang et al. (2023). The analytical method is consistent with the previous study. Briefly, the reads counts matrix was used to identify differentially expressed genes (DEGs) by the DESeq2 package. DEGs with |log2FoldChange| > 1, and P-value <0.01 were considered as significant DEGs. The TPM matrix was used to compare the expression before and after treatment in each group.

### Immunohistochemistry

Immunohistochemical (IHC) staining was performed on tumor specimens from ESCC patients after neoadjuvant therapy, specifically on formalin-fixed, paraffin-embedded (FFPE) tissue sections following a standardized protocol. Briefly, 4–5 μm thick sections were deparaffinized in xylene, rehydrated through graded ethanol series, and subjected to heat-induced epitope retrieval in citrate buffer (pH 6.0) at 95°C–100°C for 20 min. After cooling and rinsing in PBS, endogenous peroxidase activity was blocked with 3% hydrogen peroxide for 10 min. Non-specific binding was blocked with 5% normal serum for 30 min, followed by incubation with primary antibody (S100A4: Proteintech, Cat No. 16105-1-AP; α-SMA: Proteintech, Cat No. 14395-1-AP) overnight at 4°C. After PBS washes, sections were incubated with HRP-conjugated secondary antibody (Proteintech, Cat No. SA00001-2) for 1 h at room temperature. The immunoreactivity was visualized using DAB substrate, with staining intensity monitored microscopically. Sections were then counterstained with hematoxylin, dehydrated, cleared in xylene, and mounted with permanent medium. Appropriate positive and negative controls were included in each experiment. Stained sections were examined under a light microscope, and images were captured using a digital imaging system for subsequent quantitative analysis. All procedures involving human tissues were approved by the Institutional Review Board, and informed consent was obtained from all participants.

### Isolation and culturing of CAFs

Fresh esophageal carcinoma tissues were obtained from surgical specimens with informed consent and institutional ethical approval. The tissues were washed thoroughly in sterile PBS containing 1% penicillin-streptomycin (Gibco) to remove blood contaminants. After mincing into 1–2 mm^3^ fragments, the tissue pieces were digested in collagenase type I (2 mg/mL, Sigma) and hyaluronidase (100 μg/mL, Sigma) dissolved in DMEM/F12 medium at 37°C for 2–4 h with gentle agitation. The digested suspension was filtered through 100-μm and subsequently 70-μm cell strainers (Corning) to obtain single-cell suspensions. After centrifugation at 300 *g* for 5 min, the pellet was resuspended in complete growth medium (DMEM/F12 supplemented with 10% fetal bovine serum (FBS, Gibco), 1% L-glutamine, and 1% antibiotic-antimycotic solution). Cells were seeded into collagen I-coated culture flasks (Corning) and maintained at 37°C in a humidified 5% CO_2_ incubator. Non-adherent cells were removed after 24 h by medium replacement. CAFs were selectively expanded based on their rapid adhesion capability and typical spindle-shaped morphology. Cells between passages 3–6 were used for subsequent experiments to ensure phenotypic stability. The expression level of S100A4 in conditioned medium from cancer-associated fibroblasts was quantified by ELISA, yielding values ranging from 30 to 70 ng/mL.

### Culture of ESCC cell line

The KYSE150 cell line (ESCC cell line) was obtained from the cell bank of the Chinese Academy of Sciences. Cells were cultured at 37°C in a humidified atmosphere containing 5% CO_2_ and maintained in RPMI-1640 medium supplemented with 10% fetal bovine serum (FBS) and 10,000 U/mL penicillin-streptomycin. 2ug/mL of recombinant S100A4 protein (hrS100A4, MCE, HY-P71140, United States) or conditioned medium of CAF (CAF-CM) was added to the culture medium and WB assay was performed after 24 h to detect PD-L1 expression.

### Western blot

Following the 24-h treatment, tumor cells were lysed using RIPA buffer supplemented with protease and phosphatase inhibitors. The lysates were centrifuged at 12,000 × g for 15 min at 4°C, and the supernatants were collected. Protein concentration was determined using a BCA assay. Equal amounts of protein (20–50 µg) were mixed with 4× Laemmli buffer, boiled at 95°C for 5 min, and loaded onto a 10% SDS-polyacrylamide gel. Electrophoresis was performed at 100 V for 1.5–2 h in running buffer (25 mM Tris, 192 mM glycine, 0.1% SDS). Proteins were transferred to a PVDF membrane via wet transfer at 100 V for 1.5 h in transfer buffer (25 mM Tris, 192 mM glycine, 20% methanol). The membrane was blocked with 5% non-fat milk in TBST for 1 h at room temperature, then incubated overnight at 4°C with a primary antibody against PD-L1 (Proteintech, Cat No. 66248-1-Ig) diluted in 5% BSA/TBST. After washing three times with TBST, the membrane was probed with HRP-conjugated secondary antibodies (Proteintech, Cat No. SA00001-1) for 1 h at room temperature. Protein bands were visualized using enhanced chemiluminescence substrate and imaged with a chemiluminescence detection system, with band intensity quantified using ImageJ software.

### Statistics and reproducibility

No statistical method was used to predetermine the sample size. The sample sizes of sequencing were primarily decided based on the availability of samples and previously published research articles of the same type. Detailed protocols for analyzing data and gene expression data are described in the Methods. R was applied for wilcoxon test and correlation analysis with scRNA-seq data. For experimental data, GraphPad Prism 8 was used to perform statistical analyses and graphics production. All the statistical analyses were performed in two-tailed manner. P values less than 0.05 were considered statistically significant.

## Results

### Unified single-cell maps across cancer types

**FIGURE 1:**
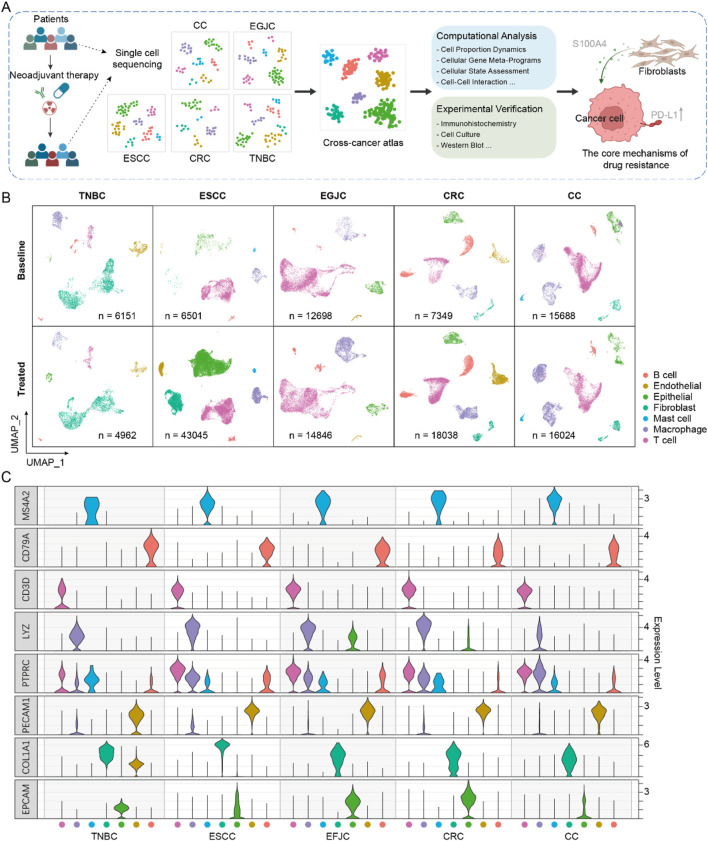
Single-cell atlas of neoadjuvant therapy in multiple cancers. **(A)** The workflow for integrating and analyzing single-cell data associated with neoadjuvant therapy across multiple cancers. **(B)** The single-cell atlas of five cancers, where cells are uniformly classified into seven major categories. **(C)** The expression profiles of marker genes in each cancer.

Single-cell RNA-sequencing datasets of tumor tissues from patients undergoing NAT with esophageal squamous cell carcinoma (ESCC), esophagogastric junction carcinoma (EGJC), colorectal cancer (CRC), cervical cancer (CC) and triple-negative breast cancer (TNBC) were obtained (Yang et al., 2023; Soto et al., 2024; Li et al., 2023; Dai et al., 2024; Jin et al., 2024) (Figure 1A). After quality control and filtering, a total of 145,302 cells across 51 samples were retained, including 48,387 cells from 19 pre-treatment samples and 96,915 cells from 32 post-treatment samples. All the data was generated by 10X Genomics single-cell RNA sequencing.

To initially assess the consistency of cell type composition across different cancer types, we clustered single-cell data separately. Then, we annotated the cell types of each cancer based on a uniform criterion (Figures 1B,C). Seven major cell types were annotated according to canonical markers, which were identified as epithelial cells (ranged from 2.7% to 44.1%), B cells (ranged from 1.4% to 24.1%), T cells (ranged from 7.7% to 68.3%), endothelial cells (ranged from 0.8% to 13.0%), fibroblasts (ranged from 0.4% to 66.41%), mast cells (ranged from 0.1% to 2.1%) and macrophage cells (ranged from 7.0% to 30.1%). We found that although no specific cell sorting was carried out in all samples during the preliminary experimental treatment, the distribution of cell subsets varied across different cancer types, which may be caused by sampling bias or cell capture efficiency from different studies. We integrated cells from all samples to generate an initial integrated map of cell clusters and annotations. By comparing the cell clustering and annotation results of each individual cancer type with the integrated map, we filtered out cells with inconsistent annotations (only cells with high confidence (score >0.8) were retained for subsequent analysis, see Methods).

### Integrative analysis across cancer types reveals changes in the cell proportion within the tumor ecosystem

**FIGURE 2:**
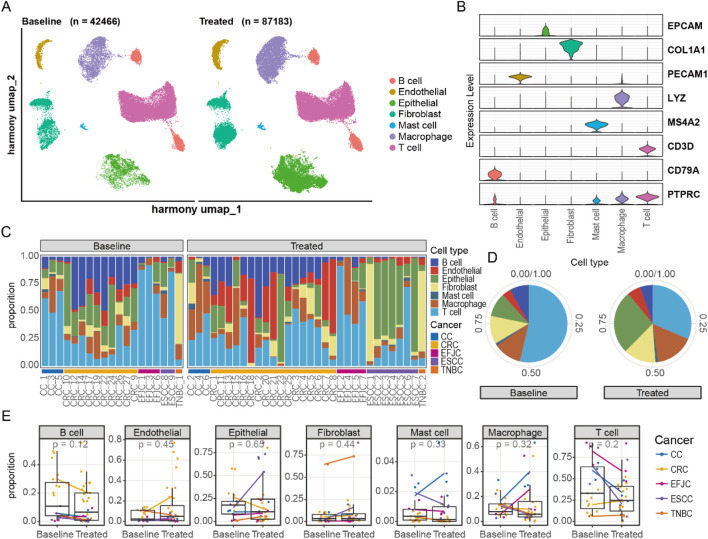
Integration of single-cell atlases of multiple cancers. **(A)** UMAP representation of changes in the integrated single-cell atlas before and after neoadjuvant therapy. **(B)** The distribution of marker genes for different cell types. **(C)** The proportion of cells in each sample. **(D)** The alterations in cell proportions before and after neoadjuvant therapy. **(E)** The changes in cell proportions before and after treatment across different cancers.

To explore the effects of neoadjuvant therapy on TME across different cancers, we integrated all remaining cells for dimensionality reduction and clustering analysis (Figure 2A; Supplementary Figure S1). The results demonstrated clear distinctions among cell types, with specific marker genes expressed exclusively within their respective clusters (Figure 2B). Additionally, the disparity in cell numbers between pre- and post-treatment samples was no longer significantly different at the order-of-magnitude level, indicating that our data preprocessing steps effectively balanced the cell counts across different samples.

We then conducted a detailed analysis of the proportion of cell types within each sample (Figure 2C). Notably, the proportion of T cells changed the most after treatment, followed by epithelial cells, indicating that neoadjuvant therapy has a strong impact on the immune system (Figure 2D). The increase in the proportion of epithelial cells was primarily observed in ESCC, highlighting the unique characteristics of esophageal epithelial cells compared to other tumors (Figure 2E). In other cell types, changes in cell proportions were similar across different cancer types. However, we observed specific changes in some cell types. For example, mast cells in cervical cancer showed an upward trend after treatment. This change may be closely related to the secretion of cervical mucus, suggesting the need to maintain a special immune environment to facilitate the migration of reproductive cells. Additionally, macrophage cells increased after treatment in CC and EGJC, potentially serving as a substitute for specific immunity to perform immune functions. In summary, the changes in cellular composition varied among different cancers, suggesting that despite similar treatment conditions, the TME exhibits marked heterogeneity between distinct cancers. This highlights the importance of considering cancer-type specificity when formulating personalized treatment plans, as cells within different cancers may exist in varying states and exert distinct functions.

### Dynamics of cellular states following neoadjuvant therapy

**FIGURE 3:**
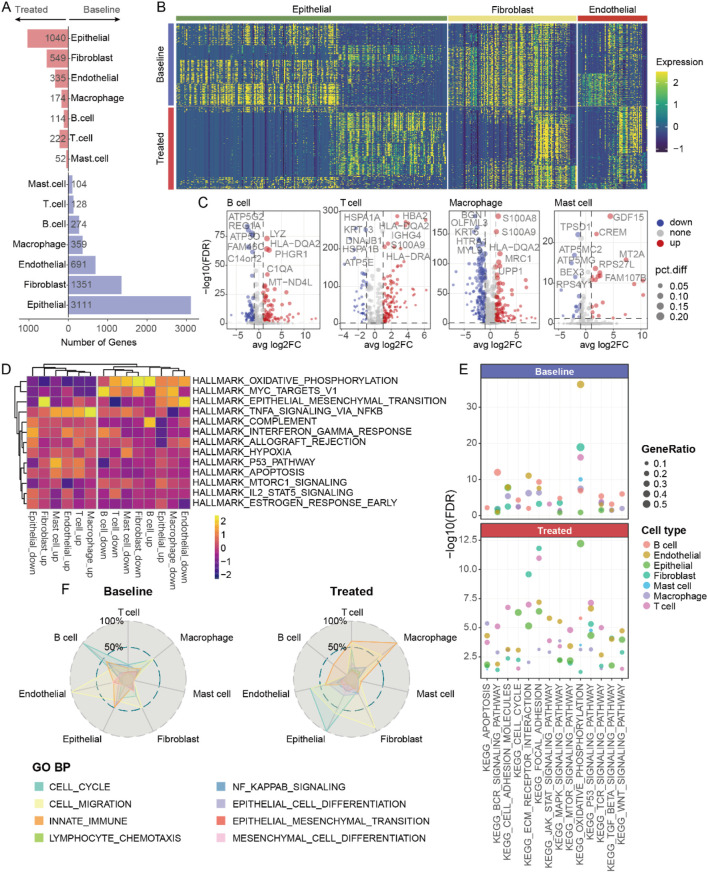
Transcriptomic changes of various cell types after neoadjuvant therapy. **(A)** The number of differentially expressed genes in different cell types before and after neoadjuvant therapy. **(B)** Heatmap showing gene expression in various non-immune cells. **(C)** Volcano plot presenting the differentially expressed genes in various immune cells. **(D)** Heatmap of hallmark analysis of differentially expressed genes in various cell types. **(E)** The pathways associated with the differentially expressed genes of various cell types. **(F)** The biological processes enriched by the differentially expressed genes of various cell types.

To elucidate the molecular changes in different cell types following neoadjuvant therapy, we conducted a detailed analysis of the alterations in gene expression patterns of seven annotated cell types (Figures 3A–C). Epithelial cells exhibited the most pronounced differences in gene expression affected by treatment. Additionally, fibroblasts and macrophage cells showed more active gene expression at baseline compared to other cell types (Figure 3B), suggesting that these cells might play a crucial role in tumor progression at baseline. Using gene set variation analysis (GSVA), we observed that fibroblasts and macrophage cells had stronger oxidative phosphorylation capabilities at baseline (Figure 3D). After neoadjuvant therapy, these cells displayed enhanced immune features, such as increased TNF, IL-2, and interferon signals. These changes indicate a shift from metabolically active to immunologically activated states during treatment. Moreover, we noted an enhancement of mTOR signaling pathways in stromal cells (endothelial and fibroblasts) and immune cells (mast, T, B, and macrophage cells) post-neoadjuvant therapy (Figure 3E). Notably, T cells showed lower levels of mTOR signaling pathway activity at baseline compared to other cell types, yet after neoadjuvant therapy, the enrichment of these pathways in T cells was significantly higher (Figure 3E). This finding further supports the notion that neoadjuvant therapy can effectively activate specific immune responses across various cancers, aiding in the clearance of tumor cells.

The activation of signaling pathways may affect the cellular state. We observed significant differences in the biological processes involved by different cell types at baseline *versus* post-treatment (Figure 3F). For example, increased endothelial cell migration at baseline likely correlated with tumor angiogenesis; whereas post-treatment, more fibroblasts underwent migratory changes, possibly associated with specific immune activation, alongside heightened innate immune function in macrophage cells. These findings indicate that changes in various cell types of post-neoadjuvant therapy are both unique and synergistic, overall presenting an enhanced immune activation profile. Changes in epithelial, fibroblast, and macrophage cells are particularly noteworthy because they likely contribute directly to treatment response and remodeling of the TME.

### Fibroblast-tumor cell interactions may generate the immunosuppressive microenvironment after treatment

**FIGURE 4:**
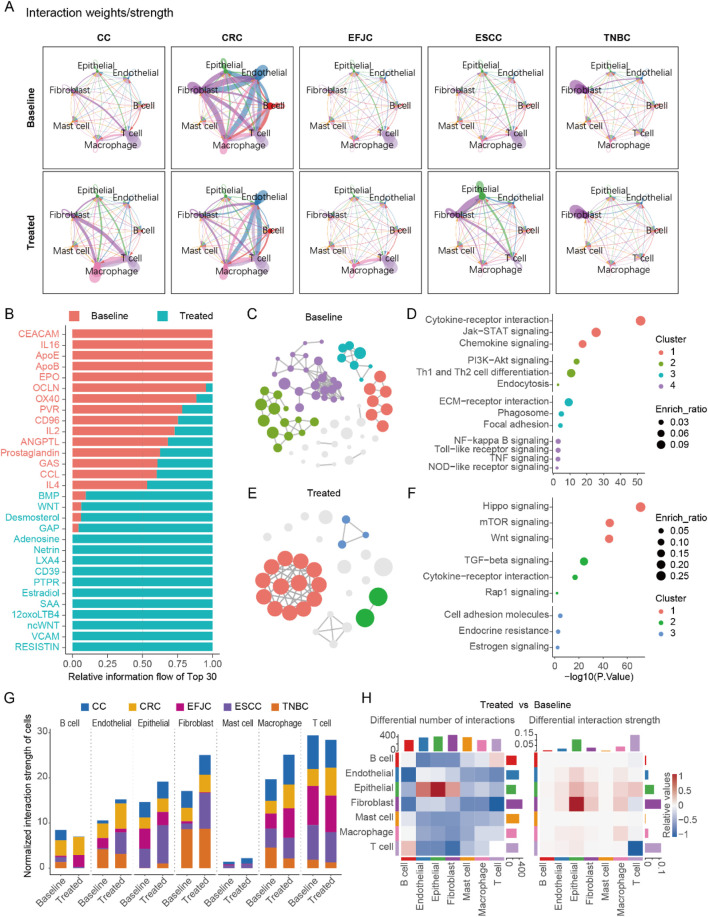
Cell-cell communication before and after neoadjuvant therapy in multi cancers. **(A)** The cell - cell communication patterns in five cancer types before and after neoadjuvant therapy. **(B)** The most significantly enriched signals, taking the top 15 at baseline and the top 15 after treatment respectively. **(C)** The pathway network of genes belonging to the significant signals at baseline in the enrichment analysis. **(D)** The pathways enriched by the significant signals at baseline. **(E)** The pathway network of genes belonging to the significant signals after treatment in the enrichment analysis. **(F)** The pathways enriched by the significant signals after treatment. **(G)** Statistical chart of cell-cell communication intensity in various cancers. **(H)** The number and intensity of cell-cell communication before and after treatment across multiple cancers.

To elucidate the alterations in intercellular communication among different cell types, we utilized the CellChat R package to analyze cell interactions across five cancers, both pre- and post-treatment (Jin et al., 2025). Our analysis revealed significant differences in cell communication patterns among the various cancers (Figure 4A). Specifically, T cell activity was notably higher in untreated CC, EFJC, and ESCC, but in untreated CRC, fibroblasts dominated. Interestingly, this fibroblast-dominant pattern was commonly observed in multiple cancers post-treatment (Figure 4A), suggesting a potential link between fibroblasts and the efficacy of neoadjuvant therapy.

A detailed analysis of the signals, with fibroblasts as signal senders and epithelial cells as receivers, identified 143 significant signals. Among these, we pinpointed 15 signals that were significantly enriched at baseline and 15 signals that were significantly enriched post-treatment (Figure 4B). At baseline, fibroblasts primarily interacted with epithelial cells via extracellular adhesion molecules, playing roles in antigen presentation and intercellular connectivity (Figures 4C,D). Post-treatment, fibroblasts activated the proliferation pathway of epithelial cells through focal adhesions, specifically the PI3K-AKT signaling pathway, which may be a key mechanism underlying tumor cell resistance to NAT (Figures 4E,F).

Furthermore, we quantified the total interaction strength (product of interaction strength and cell count) across all cell types pre- and post-treatment (Figure 4G). Fibroblasts exhibited the most pronounced changes in total interaction strength, followed by macrophage and epithelial cells. Notably, the increased fibroblast interaction strength post-treatment correlated with marked alterations in the TME, which may drive NAT resistance. This was further supported by a significant elevation in fibroblast-to-tumor cell signal output after neoadjuvant therapy (Figure 4H), highlighting fibroblasts’ pivotal role in reshaping therapeutic responses.

### Meta-programs analysis reveal the immunosuppressive role of S100A4

**FIGURE 5:**
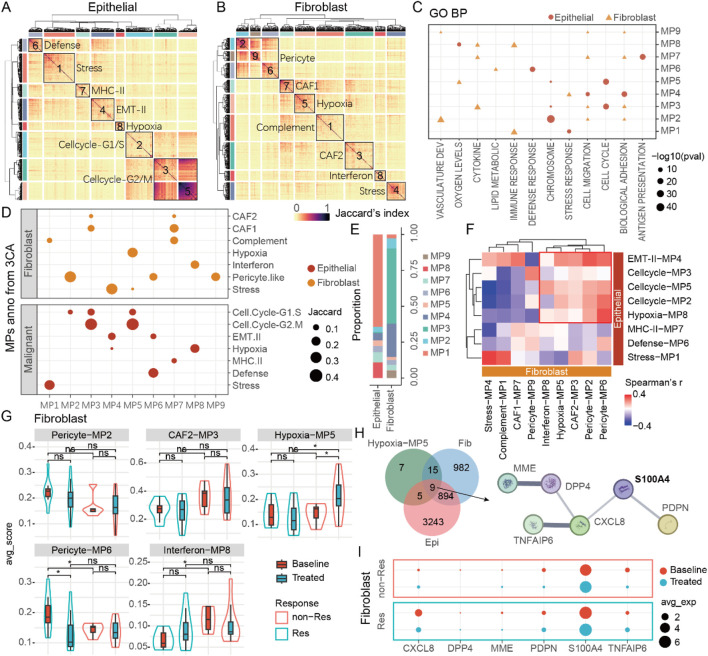
S100A4 is associated with poor efficacy and tumor progression. **(A)** The various meta-programs of epithelial cells. **(B)** The various meta-programs of fibroblasts. **(C)** GO annotation of MPs in epithelial cells and fibroblasts. **(D)** 3CA database annotation of MPs in epithelial cells and fibroblasts. **(E)** The proportions of various MPs in epithelial cells and fibroblasts. **(F)** The correlation between MPs in epithelial cells and fibroblasts. **(G)** MPs related to tumor progression in fibroblasts. **(H)** DEGs of epithelial cells/fibroblasts related to fibroblast MP5, as well as the key protein-protein interaction network. **(I)** The expression of key genes in fibroblasts, among which S100A4 has the highest expression level.

To gain deeper insights into cell types closely associated with tumor response and remodeling, we extracted subsets of epithelial, fibroblast, and macrophage cells and performed consensus non-negative matrix factorization (cNMF) analysis on these subsets. The results indicated that epithelial, fibroblast, and macrophage cells possessed seven, seven, and eight distinct cellular states, respectively (Figure 5; Supplementary Figure S2A). We annotated the different states (Meta-Programs, MPs) of each cell type using Gene Ontology Biological Process (GO BP) enrichment analysis and corroborated these findings with data from public databases of The Curated Cancer Cell Atlas (3CA) (Tyler et al., 2024) (Figures 5C,D; Supplementary Figures S2C,D). Ultimately, we defined the states of these 3 cell types, identifying that epithelial cells predominantly exhibited stress-induced states (Stress-MP1, 63.3%), fibroblasts showed a high prevalence of cancer-associated fibroblasts (CAF2-MP3, 52.4%), and several immune regulation-related functions were involved in macrophage cells (Figure 5E; Supplementary Figure S2B). Through correlation analysis of cell proportions, we identified that the cell-cycle MPs of epithelial cells was associated with five MPs in fibroblasts (Figure 5F). Further analysis of these five fibroblast MPs revealed that the fibroblast hypoxia-related MP5 might be linked to therapy resistance. Notably, while fibroblast hypoxia-related MP5 showed no baseline differences, its score significantly increased in post-treatment non-responders, suggesting its potential role in aiding tumor cells to resist therapy (Figure 5G). Additionally, we discovered nine genes co-differentially expressed in both epithelial cells and fibroblasts post-treatment that were functionally linked to fibroblast MP5. Subsequently, three criteria were applied to further analyze the nine genes: 1) retention of genes with mean expression levels in the top 50%; 2) differential expression with fold change >0.5 in ≥3 cancer types; and 3) significant involvement in protein-protein interaction networks based on StringDB analysis. Ultimately, we identified a regulatory module composed of six genes that may play a critical potential role in NAT resistance. (Figure 5H). S100A4 emerged as a key candidate among these six genes, displaying the highest expression level in fibroblasts and significant upregulation in non-responders after therapy (Figure 5I). In addition, the defense-related MP of epithelial cells are associated with the MPs of macrophage cells (Supplementary Figures S2E,F). There are significant differences in the expression of S100A4 between epithelial cells and macrophage cells (Supplementary Figure S2G). Deeper analysis indicated a positive correlation between S100A4 expression and the proportion of macrophage cells after treatment. As S100A4 levels rose, CD163 expression in macrophage also increased (Supplementary Figures S2H,I). This implies that S100A4 may play a role in the formation of an immunosuppressive TME.

### S100A4 in fibroblasts can promote PD-L1 expression in tumor cells

**FIGURE 6:**
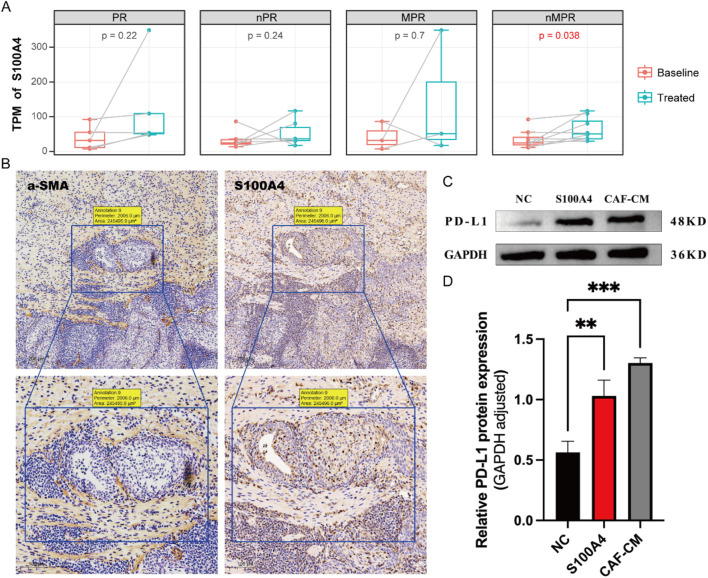
Fibroblasts released S100A4 to promote PD-L1 expression in epithelial cells. **(A)** Bulk RNA results demonstrate that S100A4 significantly rises after neoadjuvant therapy in the nMPR group. **(B)** IHC results of a-SMA (the fibroblast marker protein) and S100A4. The a-SMA and S100A4 show co-localization. **(C)** Western blot reveals the expression of PD-L1 protein under different stimulations. **(D)** Both S100A4 and the fibroblast culture medium (CAF-CM) can significantly enhance the expression of PD-L1, but there is no significant difference between S100A4 and CAF-CM.

By integrating scRNA-seq data across various cancers, we propose that S100A4 in the TME leading to tumor resistance against neoadjuvant therapy. Analyzing additional bulk RNA-seq data revealed that S100A4 expression is significantly correlated with poorer outcomes of neoadjuvant therapy (Figure 6A; Supplementary Figure S3A). Given the pivotal role of S100A4 within the fibroblast MP (CAF2-MP3), we further utilized the immunohistochemistry (IHC) assay to confirm its expression in fibroblasts. As expected, we detected a striking co-localization between α-SMA, an established fibroblast marker gene, and S100A4, indicating that fibroblasts are a significant source of S100A4 (Figure 6B). These results suggest that S100A4 is likely a key component of conditioned media from cancer-associated fibroblasts (CAF-CM).

Subsequently, we conducted further experiments to validate how CAF-CM cells confer immune resistance to tumor cells through the expression of S100A4. We discovered that both S100A4 and CAF-CM cells could stimulate tumor cells to highly express the PD-L1, a known immunosuppressive marker (Supplementary Figures S3B,C). High expression of PD-L1 can effectively inhibit the cytotoxic function of T cells, thereby reducing tumor cell apoptosis and leading to poor therapeutic outcomes (Huseni et al., 2023). Notably, we observed no significant difference in promoting PD-L1 expression on tumor cells between S100A4 and CAF-CM (Figures 6C,D), suggesting that S100A4 alone is sufficient to drive this effect. In summary, S100A4, secreted by CAFs, promotes PD-L1 expression on tumor cells, weakening the immune system’s ability to attack these cells and leading to poorer treatment outcomes.

## Discussion

In the present study, through systematic characterization of dynamic changes in tumor cells and their microenvironment following neoadjuvant therapy, combined with intercellular crosstalk analysis, we identified that interactions between fibroblasts and tumor cells represent a key determinant of neoadjuvant resistance. Further analysis revealed that post-teatment fibroblasts exhibited upregulation of the S100A4 gene and activation of immunosuppressive pathways. *In vitro* experiments demonstrated that CAF-secreted S100A4 promotes PD-L1 expression in tumor cells, uncovering a potential molecular mechanism linking S100A4 to neoadjuvant therapy resistance.

Based on unified single-cell maps across cancer types, we systematically characterized dynamic changes in the tumor ecosystem and intercellular crosstalk before and after neoadjuvant therapy to identify core mechanisms of resistance. It is noteworthy that in this study, patients in the TNBC dataset received chemotherapy only; those in the ESCC dataset were treated with chemotherapy, radiotherapy, and immunotherapy; EGJC patients received immunotherapy targeting CD40 only; CRC patients were treated with immunotherapy targeting PD-1 only; and CC patients received chemotherapy and radiotherapy. Due to the differences in neoadjuvant treatment approaches adopted for different cancer types and their subtypes according to clinical guidelines and consensus, our study has difficulty in obtaining data on uniform treatment patterns for analysis. This indeed introduce certain confounding factors that affect the analytical results and conclusions of this study. Precisely for this reason, we found that dynamic patterns of tumor cells and their microenvironment before and after treatment varied across cancer types or treatment approaches. Meanwhile, we also observed diversity in the molecular-level dynamics for each cell population. These differences might be largely attributed to variations in cancer types and treatment modalities. Notwithstanding this, we further identified conserved and robust trends across cancer types, notably the suppression of the tumor immune microenvironment following neoadjuvant therapy. Further analysis of cell-cell interactions revealed that fibroblasts played a central role in the interaction network, and this finding was not specific to a particular cancer type but exhibited cross-cancer characteristics. We subsequently further analyzed the robust cell communication capabilities of fibroblasts and the mechanisms of their immunosuppressive effects. In summary, influenced by cancer types and treatment modalities, the dynamic changes of the tumor ecosystem before and after treatment show certain differences. However, focusing on the robust findings across cancer types and treatment approaches, particularly the immunosuppressive effect centered on fibroblasts, we systematically studied the potential mechanisms by which fibroblasts act on tumor cells to induce immune escape.

Fibroblasts play a crucial role in the TME, exerting various tumorigenic functions such as extracellular matrix (ECM) remodeling and the secretion of cytokines, chemokines, and growth factors (Sarkar et al., 2023). By interacting with cancer cells, CAFs create a supportive niche for cancer stem cells, leading to the suppression of the tumor immune microenvironment and reprogramming of cancer cell metabolism, which ultimately promotes tumor metastasis and immune evasion (Liu et al., 2023). In our current study, we discovered that the CAF-CM can effectively stimulate tumor cells to express PD-L1, and high expression of PD-L1 can contribute to tumor escape. Our findings delineate a pivotal role of S100A4 secreted by CAFs, primarily by stimulating tumor cells to express PD-L1, which leads to immune escape and weakens the efficacy of neoadjuvant therapy.

Our study further found that S100A4 is expressed in macrophages, suggesting that macrophage-derived S100A4 may also play a role in suppressing the tumor immune microenvironment. A previous study reported a relationship between PD-L1 and M2 macrophages (Yamaguchi et al., 2022). Specifically, M2 macrophages can stimulate tumor cells to express PD-L1 by engaging receptors, thereby enhancing the immune escape capability of tumor cells. In our study, the expression of CD163 in macrophage increased significantly after neoadjuvant therapy. Moreover, with the increase in CD163 expression post-treatment, the expression of S100A4 was positively correlated with the proportion of macrophage cells (Supplementary Figure S2). Nevertheless, given the substantial evidence in our study indicating the importance of CAFs, we conducted an in-depth analysis of the relationship between CAFs and S100A4. This does not imply that S100A4 and macrophages are unimportant in resistance, but rather reflects the different focus of our study. Additionally, when analyzing the interaction between CAFs and epithelial cells or analyzing their co-localization, we focused only on the signal from CAFs and epithelial cells. Therefore, during data analysis and experiments, we did not incorporate macrophage-derived S100A4 into the analysis. As such, macrophage-derived S100A4 does not affect the results and conclusions of our study on the relationship between CAFs and epithelial cells.

Through single-cell data and *in vitro* experiments, we found that S100A4 did not directly promote the proliferation of tumor cells, but it could enhance the expression of PD-L1 in tumor cells, thereby increasing their immune escape capabilities. Therefore, blocking S100A4 stimulation of tumor cells may be a potential strategy to address the poor efficacy of neoadjuvant therapy.

In addition to the impact on the results caused by the differences in neoadjuvant treatment approaches for different cancer types mentioned above, our study does have several limitations that should be acknowledged. Firstly, the sample size is limited; therefore, it is imperative to conduct further validation studies involving larger cohorts. Secondly, the efficacy of neoadjuvant therapy was not documented in most patients in scRNA data, making it challenging to identify the roles of S100A4 and their contribution to the formation of an immunosuppressive TME. Thirdly, the underlying mechanism by which cancer cells promote PD-L1 expression in response to S100A4 stimulation requires further investigation.

In conclusion, our study successfully generated a single-cell atlas of multiple cancers and provides a potential mechanism to explain the poor efficacy of neoadjuvant therapy and the formation of an immunosuppressive TME. These findings are of great significance in guiding neoadjuvant therapy for cancer patients. Our findings may help develop strategies to prevent and treat cancer.

## Conclusion

This study integrates single-cell transcriptomic data across five solid tumors, uncovering a conserved resistance mechanism to nICT. CAFs upregulate S100A4, which induces PD-L1 in tumor cells, creating an immunosuppressive microenvironment. These findings enhance our understanding of stromal-epithelial crosstalk in neoadjuvant-therapy-related immune evasion. Identifying S100A4 as a PD-L1 upregulator fills knowledge gaps in treatment resistance. Translational applications are significant: targeting S100A4 or its downstream pathways may overcome immunosuppression and boost nICT efficacy. The cross-cancer atlas is a resource for precision oncology, helping find shared vulnerabilities. This work highlights stromal components’ role in treatment outcomes and supports adding stromal-targeted therapies to future neoadjuvant regimens.

## Data Availability

The original contributions presented in the study are included in the article/Supplementary Material, further inquiries can be directed to the corresponding authors.

## Abbreviations

nICT, Neoadjuvant immunochemotherapy; scRNA-seq, Single-cell RNA-sequencing; MPs, Meta-programs; TME, Tumor microenvironment; ESCC, Esophageal squamous cell carcinoma; EGJC, Esophagogastric junction carcinoma; CRC, Colorectal cancer; DEGs, Differentially expressed genes; GSEA, Gene set enrichment analysis; CAF-CM, Conditioned media from CAFs; CC, Cervical cancer; TNBC, Triple-negative breast cancer; GSVA, Gene set variation analysis; cNMF, Consensus non-negative matrix factorization; GOBP, Gene ontology biological process; 3CA, The curated cancer cell atlas; IHC, Immunohistochemistry; NAT, Neoadjuvant therapy; CAFs, Cancer-associated fibroblasts.

## References

[B1] Bagaev A. Kotlov N. Nomie K. Svekolkin V. Gafurov A. Isaeva O. (2021). Conserved pan-cancer microenvironment subtypes predict response to immunotherapy. Cancer Cell. 39 (6), 845–865.e7. 10.1016/j.ccell.2021.04.014 34019806

[B2] Canzler S. Hackermüller J. (2020). multiGSEA: a GSEA-Based pathway enrichment analysis for multi-omics data. BMC Bioinforma. 21 (1), 561. 10.1186/s12859-020-03910-x PMC7720482 33287694

[B3] Chen C. Wang Z. Ding Y. Qin Y. (2023). Tumor microenvironment-mediated immune evasion in hepatocellular carcinoma. Front. Immunol. 14, 1133308. 10.3389/fimmu.2023.1133308 36845131 PMC9950271

[B4] Dai D. Pei Y. Zhu B. Wang D. Pei S. Huang H. (2024). Chemoradiotherapy-induced ACKR2+ tumor cells drive CD8+ T cell senescence and cervical cancer recurrence. Cell. Rep. Med. 5 (5), 101550. 10.1016/j.xcrm.2024.101550 38723624 PMC11148771

[B5] Duan H. Shao C. Pan M. Liu H. Dong X. Zhang Y. (2022). Neoadjuvant pembrolizumab and chemotherapy in resectable esophageal cancer: an open-label, single-arm study (PEN-ICE). Front. Immunol. 13, 849984. 10.3389/fimmu.2022.849984 35720388 PMC9202755

[B6] Franzén O. Gan L.-M. Björkegren J. L. M. (2019). PanglaoDB: a web server for exploration of mouse and human single-cell RNA sequencing data. Database 2019, baz046. 10.1093/database/baz046 30951143 PMC6450036

[B7] Ginestet C. (2011). ggplot2: elegant graphics for data analysis. J. R. Stat. Soc. Ser. A Stat. Soc. 174 (1), 245–246. 10.1111/j.1467-985x.2010.00676_9.x

[B8] Han S. Bao X. Zou Y. Wang L. Li Y. Yang L. (2023). d-lactate modulates M2 tumor-associated macrophages and remodels immunosuppressive tumor microenvironment for hepatocellular carcinoma. Sci. Adv. 9 (29), eadg2697. 10.1126/sciadv.adg2697 37467325 PMC10355835

[B9] Hao Y. Hao S. Andersen-Nissen E. Mauck W. M. Zheng S. Butler A. (2021). Integrated analysis of multimodal single-cell data. Cell. 184 (13), 3573–3587.e29. 10.1016/j.cell.2021.04.048 34062119 PMC8238499

[B10] Hao Y. Stuart T. Kowalski M. H. Choudhary S. Hoffman P. Hartman A. (2024). Dictionary learning for integrative, multimodal and scalable single-cell analysis. Nat. Biotechnol. 42 (2), 293–304. 10.1038/s41587-023-01767-y 37231261 PMC10928517

[B11] Hu C. Li T. Xu Y. Zhang X. Li F. Bai J. (2023). CellMarker 2.0: an updated database of manually curated cell markers in human/mouse and web tools based on scRNA-seq data. Nucleic Acids Res. 51 (D1), D870–D876. 10.1093/nar/gkac947 36300619 PMC9825416

[B12] Huang Y. Sun J. Li J. Zhu D. Dong M. Dou S. (2023). Neoadjuvant immunochemotherapy for locally advanced resectable oral squamous cell carcinoma: a prospective single-arm trial (illuminate trial). Int. J. Surg. Lond Engl. 109 (8), 2220–2227. 10.1097/JS9.0000000000000489 PMC10442116 37288582

[B13] Huseni M. A. Wang L. Klementowicz J. E. Yuen K. Breart B. Orr C. (2023). CD8+ T cell-intrinsic IL-6 signaling promotes resistance to anti-PD-L1 immunotherapy. Cell. Rep. Med. 4 (1), 100878. 10.1016/j.xcrm.2022.100878 36599350 PMC9873827

[B14] Jiang S. Liu Y. Zheng H. Zhang L. Zhao H. Sang X. (2023). Evolutionary patterns and research frontiers in neoadjuvant immunotherapy: a bibliometric analysis. Int. J. Surg. Lond Engl. 109 (9), 2774–2783. 10.1097/JS9.0000000000000492 PMC10498839 37216225

[B15] Jin H. Chen Y. Zhang D. Lin J. Huang S. Wu X. (2024). YTHDF2 favors protumoral macrophage polarization and implies poor survival outcomes in triple negative breast cancer. iScience 27 (6), 109902. 10.1016/j.isci.2024.109902 38812540 PMC11134561

[B16] Jin S. Plikus M. V. Nie Q. (2025). CellChat for systematic analysis of cell-cell communication from single-cell transcriptomics. Nat. Protoc. 20 (1), 180–219. 10.1038/s41596-024-01045-4 39289562

[B17] Kanani A. Veen T. Søreide K. (2021). Neoadjuvant immunotherapy in primary and metastatic colorectal cancer. Br. J. Surg. 108 (12), 1417–1425. 10.1093/bjs/znab342 34694371 PMC10364874

[B18] Kanehisa M. Furumichi M. Tanabe M. Sato Y. Morishima K. (2017). KEGG: new perspectives on genomes, pathways, diseases and drugs. Nucleic Acids Res. 45 (D1), D353–D361. 10.1093/nar/gkw1092 27899662 PMC5210567

[B19] Korsunsky I. Millard N. Fan J. Slowikowski K. Zhang F. Wei K. (2019). Fast, sensitive and accurate integration of single-cell data with harmony. Nat. Methods 16 (12), 1289–1296. 10.1038/s41592-019-0619-0 31740819 PMC6884693

[B20] Li J. Wu C. Hu H. Qin G. Wu X. Bai F. (2023). Remodeling of the immune and stromal cell compartment by PD-1 blockade in mismatch repair-deficient colorectal cancer. Cancer Cell. 41 (6), 1152–1169.e7. 10.1016/j.ccell.2023.04.011 37172580

[B21] Li Y. Jiang M. Aye L. Luo L. Zhang Y. Xu F. (2024). UPP1 promotes lung adenocarcinoma progression through the induction of an immunosuppressive microenvironment. Nat. Commun. 15 (1), 1200. 10.1038/s41467-024-45340-w 38331898 PMC10853547

[B22] Liberzon A. Birger C. Thorvaldsdóttir H. Ghandi M. Mesirov J. P. Tamayo P. (2015). The molecular signatures database (MSigDB) hallmark gene set collection. Cell. Syst. 1 (6), 417–425. 10.1016/j.cels.2015.12.004 26771021 PMC4707969

[B23] Liu Y. Xun Z. Ma K. Liang S. Li X. Zhou S. (2023). Identification of a tumour immune barrier in the HCC microenvironment that determines the efficacy of immunotherapy. J. Hepatol. 78 (4), 770–782. 10.1016/j.jhep.2023.01.011 36708811

[B24] Luo Q. Dong Z. Xie W. Fu X. Lin L. Zeng Q. (2023). Apatinib remodels the immunosuppressive tumor ecosystem of gastric cancer enhancing anti-PD-1 immunotherapy. Cell. Rep. 42 (5), 112437. 10.1016/j.celrep.2023.112437 37097818

[B25] Manabe T. Bivona T. G. (2022). Remodeling of the tumor/tumor microenvironment ecosystem during KRAS G12C inhibitor clinical resistance in lung cancer. J. Clin. Investig. 132 (4), e156891. 10.1172/JCI156891 35166243 PMC8843703

[B26] Mao X. Xu J. Wang W. Liang C. Hua J. Liu J. (2021). Crosstalk between cancer-associated fibroblasts and immune cells in the tumor microenvironment: new findings and future perspectives. Mol. Cancer 20 (1), 131. 10.1186/s12943-021-01428-1 34635121 PMC8504100

[B27] McInnes L. Healy J. Melville J. (2020). UMAP: uniform manifold approximation and projection for dimension reduction.

[B28] Ontology Consortium G. Aleksander S. A. Balhoff J. Carbon S. Cherry J. M. Drabkin H. J. (2023). The gene ontology knowledgebase in 2023. Genetics 224 (1), iyad031. 10.1093/genetics/iyad031 36866529 PMC10158837

[B29] Sarkar M. Nguyen T. Gundre E. Ogunlusi O. El-Sobky M. Giri B. (2023). Cancer-associated fibroblasts: the chief architect in the tumor microenvironment. Front. Cell. Dev. Biol. 11, 1089068. 10.3389/fcell.2023.1089068 36793444 PMC9923123

[B30] Sathe A. Mason K. Grimes S. M. Zhou Z. Lau B. T. Bai X. (2023). Colorectal cancer metastases in the liver establish immunosuppressive spatial networking between tumor-associated SPP1+ macrophages and fibroblasts. Clin. Cancer Res. Off. J. Am. Assoc. Cancer Res. 29 (1), 244–260. 10.1158/1078-0432.CCR-22-2041 PMC9811165 36239989

[B31] Soto M. Filbert E. L. Yang H. Starzinski S. Starzinski A. Gin M. (2024). Neoadjuvant CD40 agonism remodels the tumor immune microenvironment in locally advanced esophageal/gastroesophageal junction cancer. Cancer Res. Commun. 4 (1), 200–212. 10.1158/2767-9764.CRC-23-0550 38181044 PMC10809910

[B32] Subramanian A. Tamayo P. Mootha V. K. Mukherjee S. Ebert B. L. Gillette M. A. (2005). Gene set enrichment analysis: a knowledge-based approach for interpreting genome-wide expression profiles. Proc. Natl. Acad. Sci. 102 (43), 15545–15550. 10.1073/pnas.0506580102 16199517 PMC1239896

[B33] Sun Y.-Q. Zhong Q. Lv C.-B. Zhu J.-Y. Lin G.-T. Zhang Z.-Q. (2024). The safety and efficacy of neoadjuvant immunochemotherapy following laparoscopic gastrectomy for gastric cancer: a multicentre real-world clinical study. Int. J. Surg. Lond Engl. 110 (8), 4830–4838. 10.1097/JS9.0000000000001468 PMC11326023 38652275

[B34] Szklarczyk D. Kirsch R. Koutrouli M. Nastou K. Mehryary F. Hachilif R. (2023). The STRING database in 2023: protein-protein association networks and functional enrichment analyses for any sequenced genome of interest. Nucleic Acids Res. 51 (D1), D638–D646. 10.1093/nar/gkac1000 36370105 PMC9825434

[B35] Tyler M. Gavish A. Barbolin C. Tschernichovsky R. Hoefflin R. Mints M. (2024). The curated cancer cell atlas: comprehensive characterisation of tumours at single-cell resolution. 11.617836. 10.1038/s43018-025-00957-8 40341230

[B36] Wu T. Hu E. Xu S. Chen M. Guo P. Dai Z. (2021). clusterProfiler 4.0: a universal enrichment tool for interpreting omics data. Innov. Camb Mass 2 (3), 100141. 10.1016/j.xinn.2021.100141 PMC8454663 34557778

[B37] Xiao Y. Yu D. (2021). Tumor microenvironment as a therapeutic target in cancer. Pharmacol. Ther. 221, 107753. 10.1016/j.pharmthera.2020.107753 33259885 PMC8084948

[B38] Yamaguchi Y. Gibson J. Ou K. Lopez L. S. Ng R. H. Leggett N. (2022). PD-L1 blockade restores CAR T cell activity through IFN-γ-regulation of CD163+ M2 macrophages. J. Immunother. Cancer 10 (6), e004400. 10.1136/jitc-2021-004400 35738799 PMC9226933

[B39] Yang E. Wang X. Gong Z. Yu M. Wu H. Zhang D. (2020). Exosome-mediated metabolic reprogramming: the emerging role in tumor microenvironment remodeling and its influence on cancer progression. Signal Transduct. Target Ther. 5 (1), 242. 10.1038/s41392-020-00359-5 33077737 PMC7572387

[B40] Yang Y. Li Y. Yu H. Ding Z. Chen L. Zeng X. (2023). Comprehensive landscape of resistance mechanisms for neoadjuvant therapy in esophageal squamous cell carcinoma by single-cell transcriptomics. Signal Transduct. Target Ther. 8 (1), 298. 10.1038/s41392-023-01518-0 37563120 PMC10415278

[B41] Yerly L. Andreatta M. Garnica J. Domizio J. D. Gilliet M. Carmona S. J. (2024). Wounding triggers invasive progression in human basal cell carcinoma, 31.596823

[B42] Zhang G. Yuan J. Pan C. Xu Q. Cui X. Zhang J. (2023). Multi-omics analysis uncovers tumor ecosystem dynamics during neoadjuvant toripalimab plus nab-paclitaxel and S-1 for esophageal squamous cell carcinoma: a single-center, open-label, single-arm phase 2 trial. eBioMedicine 90, 104515. 10.1016/j.ebiom.2023.104515 36921563 PMC10024111

[B43] Zheng Y. Feng B. Chen J. You L. (2023). Efficacy, safety, and survival of neoadjuvant immunochemotherapy in operable non-small cell lung cancer: a systematic review and meta-analysis. Front. Immunol. 14, 1273220. 10.3389/fimmu.2023.1273220 38106421 PMC10722296

