# Supplementary material for: Deciphering the tumor ecosystem dynamics undergoing immunochemotherapy therapy across multiple cancer types unveils the immunosuppressive role of S100A4 in fibroblasts by promoting PD-L1 expression in tumor cells
Source: Front Cell Dev Biol. 2025 Jul 23;13:1613296. doi: 10.3389/fcell.2025.1613296 (PMC12325385; doi:10.3389/fcell.2025.1613296)
Supplement: Supplementary file 5 [file DataSheet1.docx]

**Supplementary Figures**


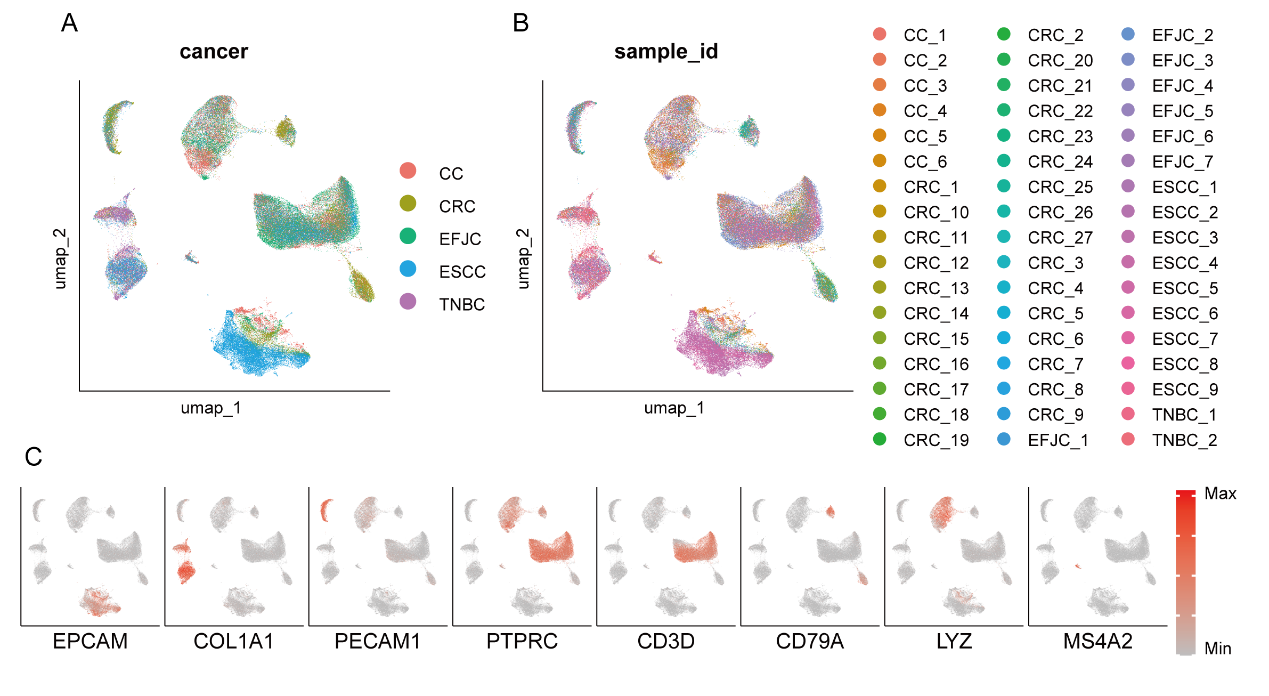


**Supplemental Figure 1** Integration of single-cell data from five cancer types

**(A)** UMAP plot of integrated single-cells in multiple cancers. **(B)** The distribution of samples in the integrated single-cell atlas. **(C)** The distribution of cell marker genes.


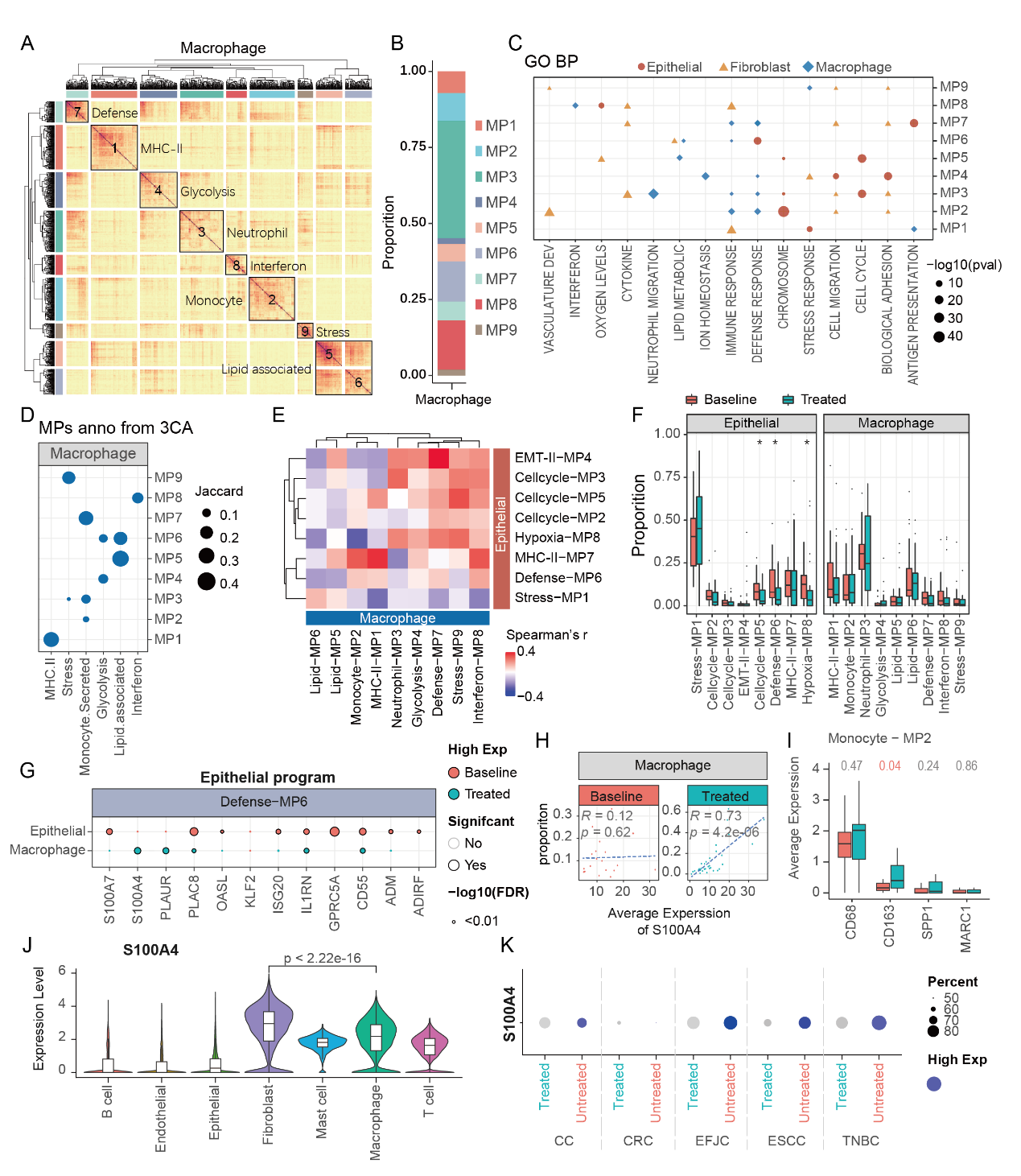


**Supplemental Figure 2** Relationship between macrophage cell MPs and neoadjuvant therapy

**(A)** The heatmap of meta-programs in macrophage cells. **(B)** The proportions of various MPs in macrophage cells. **(C)** Enrichment of biological processes for macrophage cell MPs. **(D)** 3CA annotation of MPs in myeloid cells. **(E)** The correlation between MPs in macrophage cells and epithelial cells. **(F)** The proportions of various MPs before and after treatment. **(G)** The expression of genes related to epithelial MP6 in epithelial cells and macrophage cells. **(H)** The association between the proportion of macrophage cells and the expression level of S100A4. **(I)** The changes in genes related to macrophage cell MP2 before and after treatment. The correlation analysis using the Spearman rank correlation coefficient, and the correlation coefficient and P-value are marked in the figure legend.


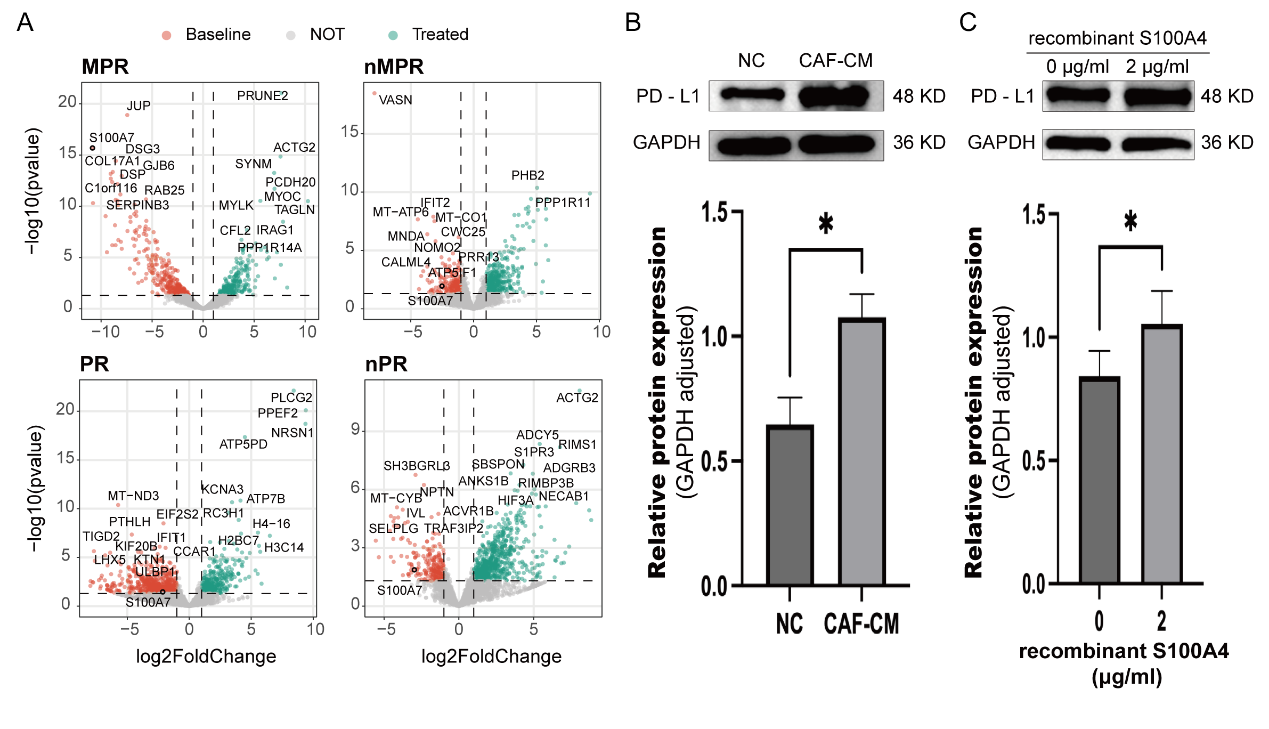


**Supplemental Figure 3** S100A4 promotes the expression of PD-L1

**(A)** The changes in Bulk RNA transcriptomes of ESCC patients before and after treatment in different groups. **(B)** CAF cells culture medium stimulates the expression of PD - L1 protein in ESCC cell lines. **(C)** S100A4 stimulates the expression of PD - L1 protein in ESCC cell lines.
